# The Immune Landscape and Molecular Subtypes of Pediatric Crohn’s Disease: Results from In Silico Analysis

**DOI:** 10.3390/jpm13040571

**Published:** 2023-03-23

**Authors:** Shiyu Xiao, Wenhui Xie, Yinghui Zhang, Yan Pan, Lei Lei

**Affiliations:** 1 Department of Gastroenterology, Sichuan Academy of Medical Sciences & Sichuan Provincial People’s Hospital, School of Medicine, University of Electronic Science and Technology of China, Chengdu 610072, China; 2 School of Medicine, University of Electronic Science and Technology of China, Chengdu 100034, China; 3 Department of Rheumatology and Clinical Immunology, Peking University First Hospital, Beijing 100034, China

**Keywords:** pediatric Crohn’s disease, immune landscape, molecular subtypes, weighted gene-co-expression network analysis, in silico analysis

## Abstract

Pediatric Crohn’s disease (CD) presents a distinct phenotype from adult-onset disease. A dysregulated immune response is critical in CD pathogenesis; thus, it is clinically important to describe immune cell alterations and to identify a new molecular classification for pediatric CD. To this end, in this study, a RNA-seq derived dataset GSE101794—which contains the expression profiles of 254 treatment-naïve pediatric CD samples, including CIBERSORTx and weighted gene-co-expression network analysis (WGCNA)—were performed to estimate the ratio of immune cells and to identify modules and genes related to specific immune cell infiltration, respectively. Hub genes derived from WGCNA were further employed to create a molecular classification using unsupervised K-means clustering. In the pediatric CD samples, it was found that M2 macrophages, CD4^+^ memory resting T cells, CD8^+^ T cells, and resting mast cells were the most prominent immune cells in intestinal tissues. Then, 985 up-regulated genes and 860 down-regulated genes were identified in samples with high immune cell infiltration. Of these differential genes, 10 hub genes (APOA1, CYB5A, XPNPEP2, SLC1A7, SLC4A6, LIPE, G6PC, AGXT2, SLC13A1, and SOAT2) were associated with CD8^+^T cell infiltration. Clinically, the higher expression of these 10 hub genes was strongly associated with an earlier age of CD onset and colonic-type CD. Furthermore, based on these key genes, pediatric CD could be classified into three molecular subtypes, displaying a different immune landscape. Altogether, this in silico analysis provides a novel insight into the immune signature of pediatric CD, and a new classification of pediatric CD is presented, which may help us develop more personalized disease management and treatments for pediatric CD.

## 1. Introduction

Crohn’s disease (CD), one of the two main forms of inflammatory bowel disease (IBD), can cause segmental and transmural damage in any section of the entire digestive tract. The incidence of CD has been reported to be increasing around the world in both adult and children populations [1]. Childhood-onset CD presents a distinct phenotype from adult-onset or elderly-onset disease in regard to its epidemiological association, clinical phenotype, and natural history [2]. The life-long nature of this disease also poses a great challenge in its treatment, especially among children. Therefore, it is clinically significant to clarify the cellular and molecular mechanisms behind CD pathogenesis and discover potential biomarkers to guide precise treatment.

While substantial progress has been made over the past few decades, a comprehensive understanding of CD pathogenesis is still out of reach. We do know that environmental, genetic, and microbial factors interact with the immune system, resulting in a dysregulated immune response responsible for the chronic intestinal inflammation of CD [3]. Immune responses are mediated by a variety of cell types, including classical immune cells, such as macrophages, natural killer cells, and T cells, as well as nonimmune cells, such as epithelial, endothelial, and mesenchymal cells [4]. In this way, a comprehensive elucidation of the key components and cellular states of the intestinal mucosal immune systems might be helpful to acquire a more in-depth understanding of the immunopathogenesis of this disease. More recently, single-cell RNA sequencing (scRNA-seq) was applied to characterize cellular heterogeneity and states in IBD [5,6]. However, for the time being, scRNA-seq remains too expensive and laborious to use on a large-sample cohort, and it cannot be applied to fixed samples collected in routine clinical practice. To the contrary, techniques for gene expression profiling, such as RNA sequencing (RNA-seq) and microarrays, have been developed and optimized to be applicable in more extensive clinical settings, resulting in the availability of a plethora of transcriptomics datasets derived from human samples, including IBD [7,8]. Using specific computational techniques, such as Cell-type Identification by Estimating Relative Subsets of RNA Transcripts (CIBERSORTx) [9], transcriptomics data can furnish information on specific cellular compositions from heterogeneous samples [10].

On the other hand, recent studies have proposed the use of some molecular phenotypes from the perspective of genomics, microbiome, epigenetics, or non-coding RNAs in IBD patients [11]. For pediatric IBD, researchers have identified that specific gene expressions, DNA methylation status, and specific microbiotas are closely associated with disease activity, disease behavior (ulcers, structuring, and penetrating complications), and even treatment response (anti-TNF use) [11]. In cancer, molecular subtypes that impact clinical phenotypes have been documented (i.e., breast cancer [12], colorectal cancer [13], and gastric cancer [14]), which lays a foundation for individual therapy. Whether CD can also be similarly separated into subgroups and whether these molecular classes can explain disease heterogeneity remain largely unknown.

Herein, we provide an overview of immune cell compositions in intestinal mucosa from naïve pediatric CD using bioinformatic techniques based on RNA-seq data. Furthermore, we explored specific genes associated with immune cell infiltration, and we constructed molecular phenotypes based on these genes.

## 2. Methods

### 2.1. RNA Expression Dataset

**Figure 1:**
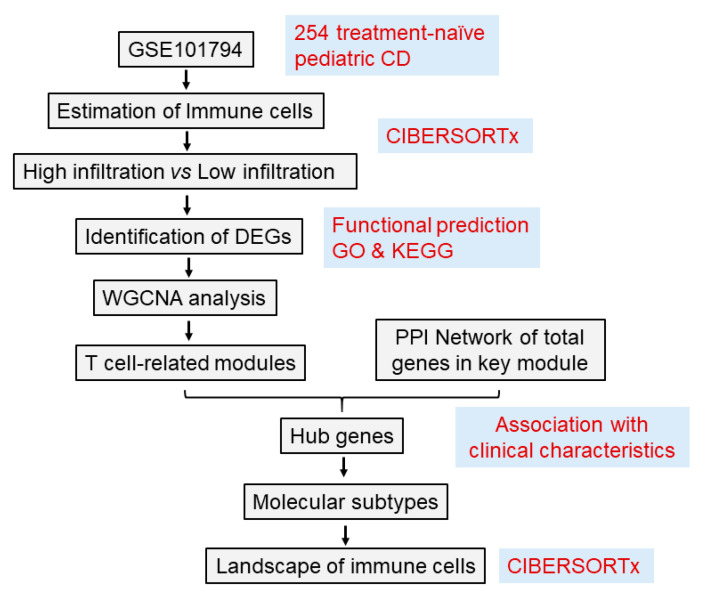
**Flow chart of study.** CD, Crohn’s disease; GO, Gene Ontology; KEGG, Kyoto Encyclopedia of Genes and Genomes; WGCNA, weight gene-co-expression network analysis; PPI, protein–protein interaction.

Dataset GSE101794 was downloaded from the Gene Expression Omnibus (GEO) database. This dataset characterizes the global gene expression profiles of ileal biopsies taken from 254 treatment-naïve pediatric CD patients and 50 non-IBD controls based on single-end, 50bp RNA-sequencing using the Illumina HiSeq2000 [15]. Notably, this dataset also furnishes information on the age-of-onset and location-of-CD of CD patients. Age of disease onset is divided into A1a (<10 years (n = 61) and A1b (10–16 years n = 193). Location of CD is grouped into L1 for ileal-only location (n = 56), L2 for colon-only location (n = 56) and L3 for ileo-colonic location (n = 142). RNA-seq data from 254 pediatric CD patients were further employed in this present analysis. Figure 1 presents an overview of the study workflow.

### 2.2. Evaluation of Tissue-Infiltrating Immune Cells Based on the RNA-Seq Data

CIBERSORTx was utilized to estimate the fraction of immune cells [9]. Briefly, the gene expression data was uploaded to the CIBERSORTx web portal, and the algorithm was run using the LM22 signature for 1000 permutations, and results with *p* < 0.05 were selected. The level of immune infiltration (high vs. low) was calculated based on the Euclidean distance and fraction of 22 immune cells.

### 2.3. Identification of Differentially Exprssed Genes from Highversus Low-Immune Cell Infiltration

The R package “limma” was employed to perform differentially expressed gene (DEG) analysis by comparing high-immune-infiltration and low-immune-infiltration (control) samples. For DEG identification, the cut-off value of |log_2_FC| > 1 and adjusted *p* < 0.05 were considered statistically significant. Then, the biological function of DEGs was analyzed by using the R package “clusterProfiler” based on Gene Ontology (GO) and Kyoto Encyclopedia of Genes and Genomes (KEGG) pathway enrichment analyses. The R package “ggplot2” was applied to show the first 10 GO results regarding biological pathway, cellular components, and molecular functions, while the package “GOplot” was used to display the first five pathways enriched in KEGG with a circus plot.

### 2.4. Construction of Gene Co-Expression Network and Identification of Hub Modules

The WGCNA algorithm in R was employed to construct a gene co-expression network of these DEGs [16]. To construct a scale-free co-expression network, an appropriate soft thresholding power value (β), ranging from 1 to 20, was first screened out. Thereafter, an adjacency matrix was built based on the Pearson’s correlation value between paired genes and transformed into a topological overlap matrix (TOM), as well as the corresponding dissimilarity (1-TOM). Next, a dynamic hybrid cutting method was applied to conduct hierarchical clustering to categorize genes with similar expression patterns into different modules. The minimum number of module genes was set to 30. Module trait associations were then estimated using the correlation between the module eigengenes and the phenotype (the infiltration level of different T cell subtypes) using the Pearson test. An individual module was considered significantly correlated with different T cell subtypes when *p* < 0.05. Finally, specific T cell subtype and modules with the highest correlation coefficient were selected, and then we defined those as the hub modules in our subsequent analysis.

### 2.5. Hub Gene Identification and Their Associations with Clinical Parameters

Candidate hub genes were first screened based on both the module membership (>0.85) and gene significance (>0.65). Module membership can be defined as the correlation of genes in the hub module and each module eigengene. Gene significance is calculated as the absolute value of the Pearson’s correlation coefficient between each gene and each trait. Meanwhile, total genes derived from the hub module were used to construct a protein–protein interaction (PPI) network to identify central nodes using the STRING (Search Tool for the Retrieval of Interacting Genes) database (https://string-db.org/ (accessed on 9 September 2022)). Central nodes were regarded as genes with node connectivity >10 and reliability >0.7. Cytoscape (https://cytoscape.org/ (accessed on 9 September 2022)) was applied to visualize the results. Venn analysis with the online tool (http://bioinformatics.psb.ugent.be/webtools/Venn/ (accessed on 9 September 2022)) was further performed to find an intersection between candidate hub genes and genes discovered in central nodes. Next, the association between hub gene expression and clinical characteristics (including age-at-diagnosis and location of CD) was evaluated. The R package “boxplot” was used to visualize the results.

### 2.6. Determination of Molecular Subtypes and Immune Characteristics

Hub genes were further employed for molecular classification based on unsupervised K-means clustering analysis. The optimal number of clusters was selected according to the sum of the squared error (SSE). The number of clusters from one to eight was tried, and the last one that significantly reduced the within groups sum of squares (at the inflection point of the curve) was selected as the optimal number of clusters. Then, sample clustering was visualized using the package “factoextra”. Finally, fractions of 22 immune cells in individual samples on the basis of the molecular subtypes were finally described using CIBERSORTx.

## 3. Results

### 3.1. Immune Landscapes of Treatment-Naïve Pediatric CD

**Figure 2:**
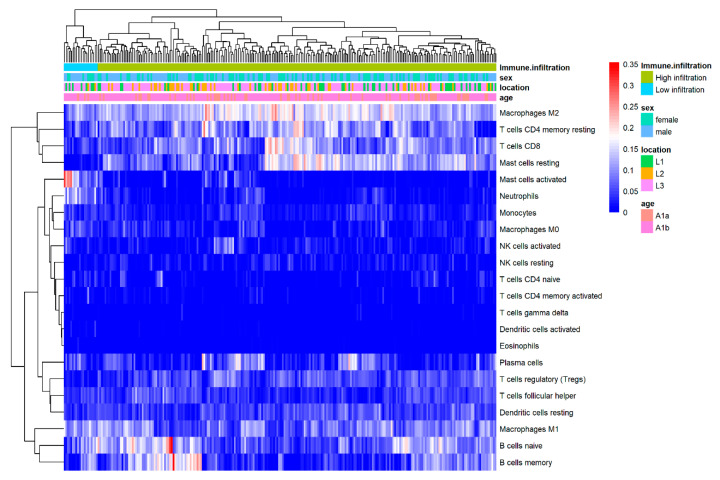
**Heatmap of the normalized absolute abundance for each immune cell type in individual samples.** Location: L1 ileal-only location, L2 colon-only location, L3 ileo-colonic location; Age: A1a < 10 years old, A1b ≥ 10 years old.

Using CIBERSORTx, the distribution of 22 immune cells within pediatric CD samples was demonstrated in a heatmap (Figure 2), revealing different fractions in the innate and adaptive immune systems. Regarding innate immune system, M2 macrophages and resting mast cells were significantly enriched, whereas neutrophils, eosinophils cells, activated mast cells, NK cells, and dendritic cells were not prominent. For the adaptive immune cells in intestine, CD8^+^ T cell, resting CD4^+^ memory T cells, naïve B cells, and memory B cells were enriched, but proportions of γδT cells, naïve CD4^+^ T cells, activated CD4^+^ memory T cells, Treg, and Tfh were low in pediatric CD samples.

### 3.2. DEGs Derived from High Immune Infiltration versus Low Immune Infiltration and Functional Analysis

**Figure 3:**
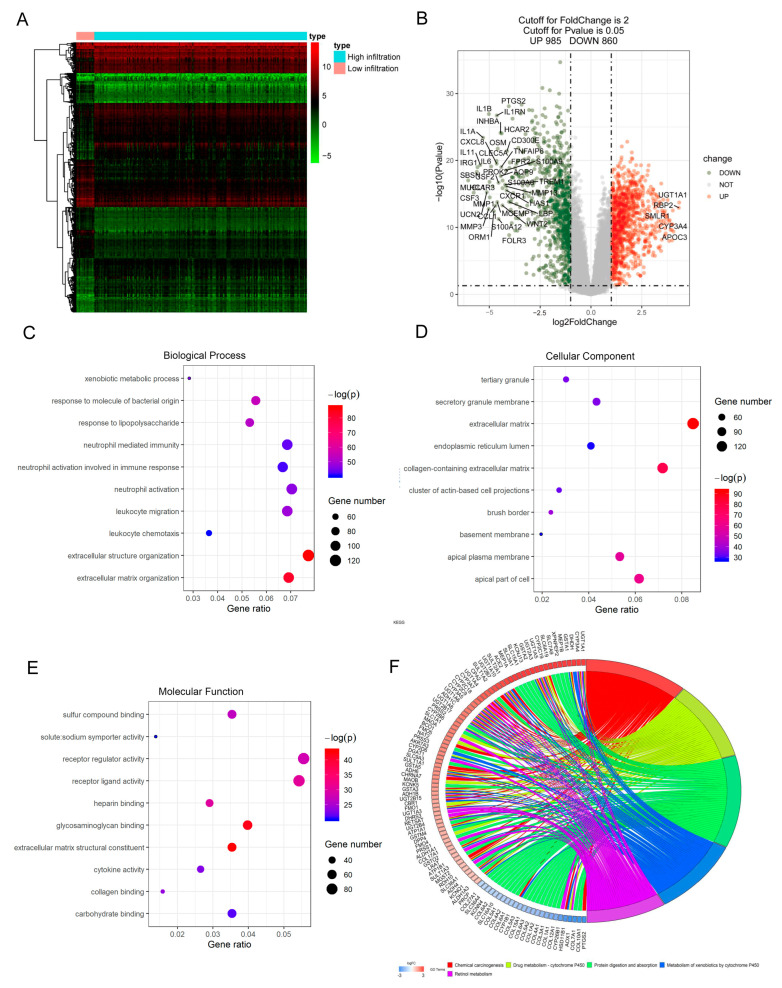
**DEGs identified from high immune cell infiltration vs. low immune cell infiltration and their functional prediction**. (**A**). Heatmap of DEGs. (**B**). Volcano plot of DEGs. Green plots represent downregulated genes, while red plots represent upregulated genes. A gene name is given when the |log_2_ FC (fold change)| > 4 and *p* < 0.01. (**C**–**E**) Enrichment of DEGs using GO analysis is visualized regarding Biological Process, Cellular Component, and Molecular Function, respectively. The larger the circle in the panel, the more genes it contains; lower *p* values are indicated with red color. (**F**). Circle plot of first five enrichment pathways for DEGs using KEGG analysis. DEGs, differentially expressed genes; GO, Gene Ontology; KEGG, Kyoto Encyclopedia of Genes and Genomes.

Based on the proportion of immune cells and Euclidean distance, we further grouped samples into high-immune-infiltration (n = 234) and low-immune-infiltration (n = 20) to obtain DEGs in samples with high immune infiltration. Finally, 1845 DEGs, including 985 upregulated genes and 860 downregulated genes, were identified in pediatric CD samples with high immune infiltration (Figure 3A,B). Functional analysis of these DEGs was performed. GO enrichment analysis revealed that DEGs were primarily related to extracellular structure organization and extracellular organization in biological processes, extracellular matrices, and apical parts of cell in the cellular component, as well as glycosaminoglycan binding and extracellular matrix structural constituent in molecular function (Figure 3C–E). KEGG enrichment analysis demonstrated that DEGs were mostly enriched in pathways related to chemical carcinogenesis, drug metabolism-cytochrome P450, protein digestion and absorption, etc. (Figure 3F).

### 3.3. Identification of Hub Modules in Relation to T Cells

**Figure 4:**
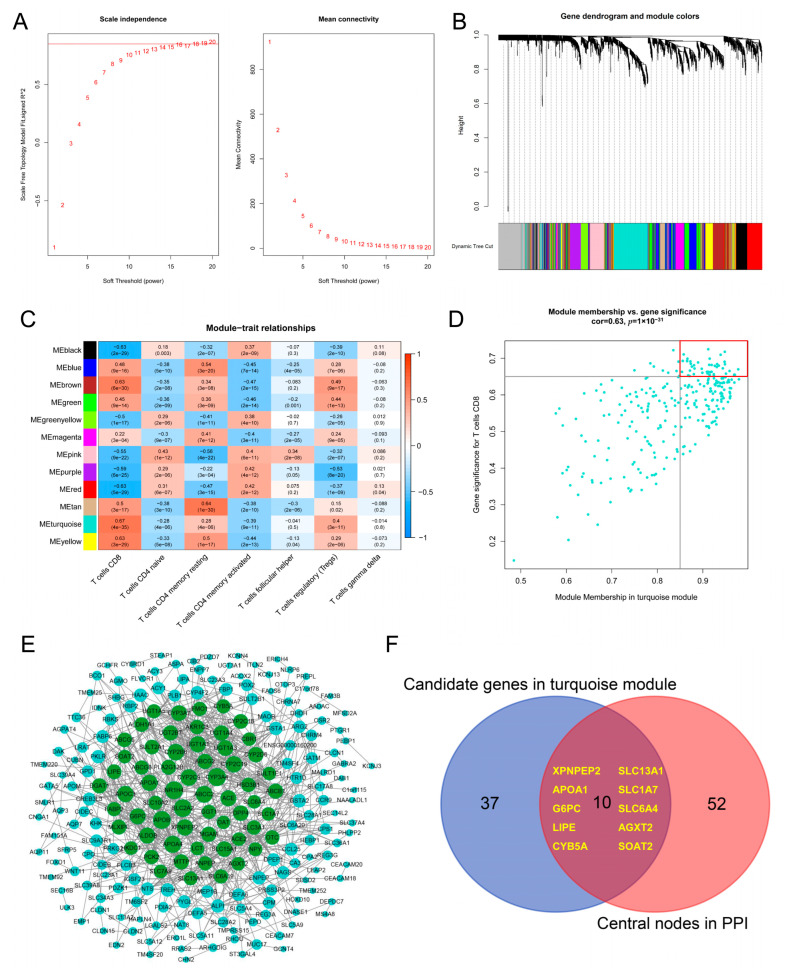
**Hub module and hub genes related to CD8^+^ T cell infiltration in pediatric CD.** (**A**). Left panel: the scale-free fit index (*y*-axis) as the function of the soft thresholding power (*x*-axis). Right panel: the mean connectivity (*y*-axis) of 1–20 soft thresholding power (*x*-axis). (**B**). The dendrogram of all DEGs clustered in different modules. (**C**). Heatmap of correlation analysis (Pearson’s correlation coefficient and *p*-value) between modules and eight T cell subtypes. (**D**). The scatter plot of each gene in the turquoise module. Dots in the red box represents genes of module membership >0.85 and gene significance >0.65. (**E**). Protein–protein interaction network of genes from the turquoise module. Nodes in green represent a central node. (**F**). Ten hub genes based on intersection between PPI and co-expression networks. WGCNA, weight gene-co-expression network analysis; PPI, protein–protein interaction.

Next, 1845 DEGs that exhibited the greatest differences between high infiltration and low infiltration were used to construct weighted co-expression networks. The optimal soft thresholding power of 16 was picked to ensure a scale-free topology (Figure 4A). A hierarchical clustering tree was then constructed using dynamic hybrid cutting, and genes with similar expression data formed a branch of the tree, representing a gene module (Figure 4B). Finally, 12 distinct gene co-expression modules were identified. Table S1 presents the number of genes in 12 modules. Regarding the significance of T cells in CD immunopathogenesis [17], the fractions of seven T cell subtypes in each sample were selected as the trait data to analyze module-trait associations. By computing the Pearson correlation coefficient with different T cell subtypes, we found that the brown (R^2^ = 0.63, *p* = 6 × 10^−30^), turquoise (R^2^ = 0.67, *p* = 4 × 10^−35^), and yellow (R^2^ = 0.63, *p* = 3 × 10^−29^) modules were highly corelated to CD8^+^ T cells, while the tan (R^2^ = 0.64, *p* = 1 × 10^−30^), blue (R^2^ = 0.54, *p* = 3 × 10^−20^), and yellow (R^2^ = 0.50, *p* = 1 × 10^−17^) modules exhibited high correlation with resting memory CD4^+^ T cells (Figure 4C). These modules were considered to be highly associated with specific T cell subtypes.

### 3.4. Hub Genes Related to CD8^+^ T Cell Infiltration in Pediatric CD

Concerning the proportion of different T cells that we presented above in pediatric CD samples and their biological function, we were specifically interested in the CD8^+^ T cells. As evident in Figure 4C, the turquoise module was highly associated with CD8^+^ T cells, indicating that connected genes in this module were potential key genes related to CD8^+^ T cell infiltration level. According to the cut-off standard (module membership > 0.85 and gene significance > 0.65), 47 genes were selected as candidate hub genes (Figure 4D). From PPI network analysis of a total of 274 genes in the turquoise module, 62 genes were identified as central nodes (genes in green in Figure 4E). Finally, 10 genes (XPNPEP2, APOA1, G6PC, LIPE, CYB5A, SLC13A1, SLC1A7, SLC6A4, AGXT2, and SOAT2) that were intersected in both analyses were designated as hub genes (Figure 4F, Table 1).

### 3.5. Associations between Hub Gene Expression and Clinical Traits

Next, we analyzed the association between the expression of hub genes and clinical parameters, including sex, age-at-diagnosis (A1a (<10 years old) and A1b (>10 years old)), and location-at-diagnosis (ileal only, colon only, or both ileal and colon). For age-at-diagnosis, we found that higher expression of these 10 hub genes was associated with earlier onset of CD (A1a subgroup) (Figure S1). Regarding disease location, a higher level of 10 hub genes was identified in colon-type (L2 location) pediatric CD (Figure S2). Lastly, we found no association between their expression level and gender difference (data not shown).

### 3.6. Identification of Molecular Subtypes and Immune Characteristics Based on Hub Genes

**Figure 5:**
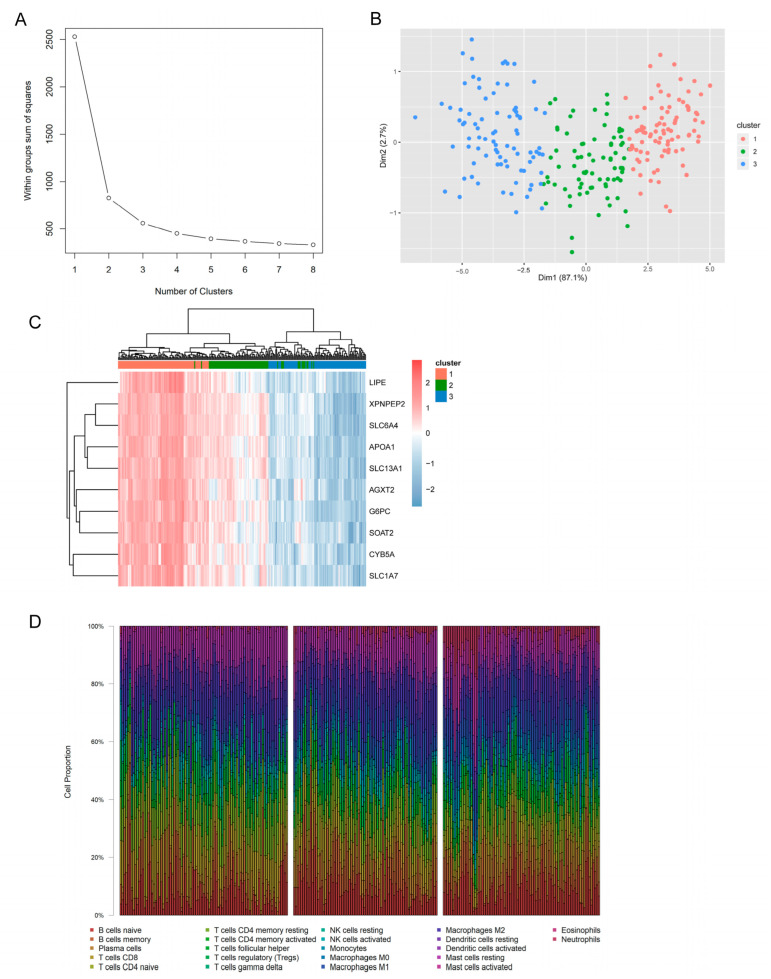
**Identification of pediatric CD subtypes.** (**A**). Identification of optimal number of clusters with non-supervised K-means clustering method. The within group sum of squares (*y*-axis) decreases with the increment of clusters number (*x*-axis). The optimal clustering was at the number of 3. (**B**). Clustering of 254 treat-naïve pediatric CD patients based on 10 hub genes. (**C**). Heatmap of 10 hub genes expression in three new subclasses of pediatric CD. (**D**). Infiltration profile of 22 immune cells in three newly proposed molecular subtypes of pediatric CD. CD, Crohn’s disease; SSE, sum of the squared error.

Based on the 10 hub genes, a new molecular subtype was constructed. It is clearly indicated that 254 naïve pediatric CD could be classified into three sub-classes (Figure 5A,B). To better clarify the three subclasses, the relationship with clinical features was assessed using Chi-square test. It was demonstrated that the proportion of samples in disease-onset age and disease location varied significantly among different subtypes (Table 2). Additionally, the expressions of these hub genes were significantly different among these three groups (Figure 5C).

Finally, we described the landscape of immune cells in these three subgroups. From the bar-plot, we found that the distribution of 22 immune cells was significantly altered among these three subgroups (Figure 5D). Specifically, pediatric CD samples in cluster 3 presented a more varied immune cell components, indicating higher infiltration of immune cells.

## 4. Discussion

Dysregulated immune response has been widely acknowledged in CD pathogenesis [18]. However, our knowledge of dysregulated immune-cell trafficking in intestinal CD, especially among children whose immune system is developing, is still lacking. In the current study, we employed bioinformatic analysis to depict the profiles of immune cells in pediatric CD and further defined a molecular subclassification based on key genes associated with specific immune cells.

Regarding the profile of immune cells, the present study demonstrated that M_2_ macrophages, CD8^+^ T cells, and resting memory CD4^+^ T cells were the most prominent immune cells in pediatric CD samples. Results from scRNA-seq also displayed an expansion of CD4^+^ T cells, myeloid cells, and IgG-producing plasma cells in the immune compartment of intestinal tissue samples collected from pediatric CD patients [19]. Macrophages are one of the classic adaptive immune cells. They have been categorized into classically activated macrophages (M_1_) or alternatively activated macrophages (M_2_) based on their cytokine secretory patterns and proinflammatory versus immunoregulatory activity [20]. Earlier studies have evidenced a role of macrophages in the pathogenesis of IBD. Dharmasiri et al. demonstrated that transcriptional profiling of macrophages from CD presented M_2_ phenotype, which was associated with fibrosis and the formation of granulomas [21]. In an animal model of trinitrobenzenesulfonic acid-induced colitis, researchers observed that the persistent M_2_-like phenotypes of macrophages and on-going intestinal stromal muscle cell proliferation might contribute to inflammatory stricture formation [22]. In this way, the enrichment of M2 macrophages in intestinal tissues from pediatric CD suggests that children with CD are more inclined to develop intestinal fibrosis and stricture in the long future. CD4^+^ and CD8^+^ T cells are two main adaptive immune cells that play a critical role in maintaining immune homeostasis. Based on evidence from animal models with spontaneous or experimentally induced colitis, CD and even ulcerative colitis (UC) have been mainly attributed to T cell-mediated inflammation, particularly mediated by CD4^+^ T cells responding to luminal antigens [3]. However, recent evidence has emphasized the role of CD8^+^ T cells as the initiators of the gut lesions and inflammatory process [23,24]. Circulating CD8^+^T cells in CD patients are capable of producing substantial proinflammatory cytokines, including IFN-γ [25]. Transcriptional profiling of circulating CD8^+^ T cells could divide CD or UC patients into two subgroups with different disease courses [26]. Another study indicated that enrichment of cytotoxic CD8^+^ T cells in the lamina propria of the neoterminal ileum coincides with CD postoperative endoscopic recurrence [27]. However, it should be noted that all of this evidence was from adult patients. Therefore, further research is necessary to elucidate the function of M_2_ macrophages and CD8^+^ T cells in pediatric CD patients.

Regarding the increasing significance of CD8^+^ T cells in gut lesion initiation, we further identified 10 hub genes derived from WGCNA analysis related to CD8^+^ T cells in pediatric CD. Some of the hub genes in our model have been reported to be involved in CD pathogenesis. APOA1, as a lipid metabolism gene, has been demonstrated to have an association with CD. Altered lipoprotein composition and associated oxidative stress have been noted in CD [28,29]. An earlier report of the global pattern of ileal gene expression in longstanding adult-onset CD has noted a profound suppression of APOA1 [30]. Haberman et al. also reported that APOA1 expression was significantly decreased in pediatric CD patients with more severe mucosal injury [31]. More importantly, functional annotation enrichment analysis of the APOA1 gene co-expression signature revealed an upregulation of an adaptive IFNG/CXCL9 Th1 signature [31]. In an animal model, APOA1 mimetic peptides could clear proinflammatory lipids from mouse intestinal tissue and plasma [32]. These studies, combined with the current study, provide further corroboration that APOA1 might be a central pathogenic gene in CD. Another hub gene we found was SLC6A4, which encodes serotonin reuptake transporter (SERT) that is responsible for transporting serotonin from the extracellular fluid to the intracellular compartment. Serotonin, also known as 5-hydroxytryptamine, is a highly preserved endogenous monoamine signaling molecule involved in various biological processes, including inflammation [33]. It is mainly synthesized in the gastrointestinal tract, principally by the enterochromaffin cells of the intestinal epithelium. A few studies have noted that SLC6A4 mRNA or SERT protein was significantly reduced in the epithelium of CD [30,34,35,36]. In colitis mice model, SERT expression was suppressed in inflamed mucosa and continuously observed with low-grade mucosal inflammation during colitis healing [35]. In line with this, studies from different groups have identified increased serotonin level in the mucosa or serum from CD patients [37] or animal models [38,39,40]. Meanwhile, serum serotonin level was highly associated with CD disease status (active, refractory, or remission) [37]. Moreover, a case–control study indicated that STin 2 VNTR polymorphism of SLC6A4 gene may contribute to CD pathogenesis [41]. Thus, this body of evidence suggests a potential mechanism behind the decreased expression of SLC6A4 and increased level of serotonin in CD. In addition to these two hub genes, decreased expression of XPNPEP2, G6PC, AGXT2, and SOAT2 in pediatric CD was also noted from ileal transcriptome analysis [31]. However, functional and mechanistic studies on their role in IBD parthenogenesis were scarce heretofore. It was identified that cell-intrinsic dimethylguanidino valeric acid generated by AGXT2 was essential for activated effector T cell survival and expansion [42]. SOAT2, which is specifically localized to enterocyte and hepatocytes, which are the critical determinants of cholesterol homeostasis [43]. Börtlein et al. reported that SOAT2 displayed a negative effect on proliferation of CD4^+^ and CD8^+^ T cells induced by T cell receptor ligation [44]. In sum, 10 key genes discovered in this study might play a critical role in modulating immune microenvironment of CD, but further research is necessary to uncover and elucidate their functions and mechanisms in CD immunopathogenesis.

Current IBD classification is primarily based on clinical presentation, including disease location, year of disease onset, and behavioral characteristics [45]. However, current clinical phenotypes for prediction of natural history are insufficient, and therapeutic response is inconsistent. In the past five years, IBD subtypes characterized by distinct molecular signature were discovered based on the profiling of genome-wide gene expression, epigenomic features, or gut microbiota compositions. A study from Weiser M’s group revealed that adult and pediatric CD could be clearly segregated into two classes (colon-like versus ileum-like), characterized by different immune responses and cellular metabolism, and these CD subclasses were associated with multiple clinical phenotypes describing disease behavior [46]. Another study revealed that a subset of CD patients expressed a unique cellular module consisting of IgG plasma cells, inflammatory mononuclear phagocytes, activated T cells, and stromal cells (named GIMATS module), which were correlated with the failure to achieve durable corticosteroid-free remission upon anti-TNF therapy [47]. In the present study, we propose a new molecular classification of pediatric CD that was related to clinical characteristics (disease-onset age and disease location) and different immune cell profiling. These 10 hub genes used for clustering also showed a negative association with the variety of immune cell infiltration, which involves patients with a varied immune cell component, and this analysis showed lower expression of these hub genes. As we discussed above, most of these hub genes were downregulated in active CD, and some were highly associated with disease status. A previous study has indicated that, compared with model that only included clinical factors, a regression model that integrated APOA1 gene expression and certain microbial abundance could more accurately predict six-month steroid-free remission [31]. In this way, we assumed that this new molecular subtype might be associated with disease activity, but further validation study is warranted in the future.

The current study has a few shortcomings that must be addressed. First, as the CD samples were biopsied from different locations of intestine, changes in immune cell landscapes of pediatric CD partially might be attributed to the variation of cell compositions in different anatomical locations. Second, we could not associate the new molecular classification with disease phenotype, disease activity status, or disease severity, owing to the lack of relative information in this dataset. Therefore, validation of this new classification in clinical values with larger set is warranted in the future. Third, because of the limitation of the dataset and the study design, we cannot conclude whether these hub genes are very specific to pediatric CD. Thus, further investigation on the role of specific hub gene in IBD pathogenesis in different age groups is necessary.

In conclusion, the present study with in silico analysis provides a novel insight into the immune signature of pediatric CD. This study defines a new classification of pediatric CD based on the gene sets related to CD8^+^ T cells. The results may deepen our understanding of immunopathogenesis in pediatric CD. A deeper understanding of these key genes discovered in this study might shed light on the discovery of potential antibodies or small molecules for targeted therapy of CD.

## Figures and Tables

**Table 1:** Information of 10 hub genes.

Gene	Degree	Full Name
XPNPEP2	23	X-prolyl aminopeptidase 2
APOA1	22	apolipoprotein A1
G6PC	21	glucose-6-phosphatase catalytic subunit 1
LIPE	15	lipase E
CYB5A	13	cytochrome b5 type A
SLC13A1	12	solute carrier family 13 member 1
SLC1A7	11	solute carrier family 1 member 7
SLC6A4	11	solute carrier family 6 member 4
AGXT2	11	alanine-glyoxylate aminotransferase 2
SOAT2	11	sterol O-acyltransferase 2

**Table 2:** Clinical characteristics of patients with distinct classification.

		Total	Cluster 1	Cluster 2	Cluster 3	Chi-Square	*p* Value
		n = 254	n = 91	n = 78	n = 85		
Sex	Male	149	52	45	52	0.338	0.844
	Female	105	39	33	33		
Age	A1a	61	28	23	10	10.545	0.005
	A1b	193	63	55	75		
Location	L1	56	16	20	20	12.380	0.015
	L2	56	31	13	12		
	L3	142	44	45	53		

## Data Availability

All data supporting the findings of this study are available within the article and its supplementary information files or from the corresponding author on reasonable request.

## Supplementary Materials

The following supporting information can be downloaded at: https://www.mdpi.com/article/10.3390/jpm13040571/s1, Table S1 The number of genes in the 12 modules; Figure S1. The association between the expression of 10 hub genes and age-of-onset of pediatric CD patients.; Figure S2. Expression of 10 hub genes regarding the location-at-diagnosis of pediatric CD.
