# Supplementary material for: The Immune Landscape and Molecular Subtypes of Pediatric Crohn’s Disease: Results from In Silico Analysis
Source: J Pers Med. 2023 Mar 23;13(4):571. doi: 10.3390/jpm13040571 (PMC10142949; doi:10.3390/jpm13040571)
Supplement: Supplementary file 1 [file jpm-13-00571-s001.zip › jpm-2169368-supplementary.pdf]

# *Supplementary Material*

## Supplementary Table

**Table S1 The number of genes in the 12 modules.**

| Module | No. of Genes |
|---|---|
| black | 112 |
| blue | 223 |
| brown | 196 |
| green | 148 |
| greenyellow | 56 |
| megenta | 90 |
| pink | 105 |
| purple | 83 |
| red | 133 |
| tan | 47 |
| turquoise | 274 |
| yellow | 161 |

## Supplementary Figures

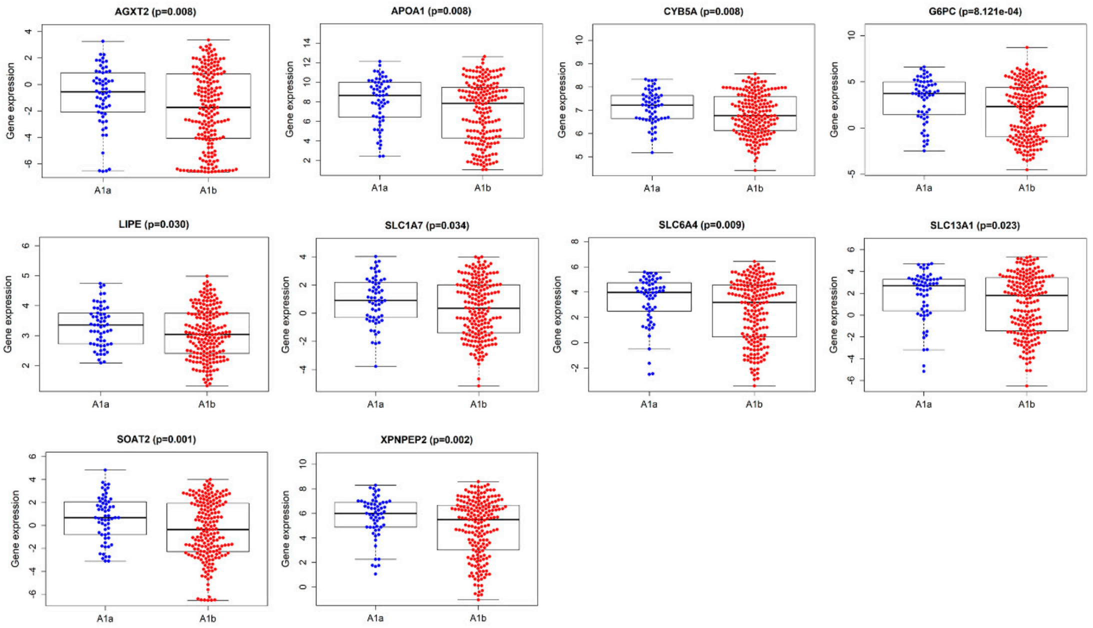


**Figure S1. The association between the expression of 10 hub genes and age-of-onset of pediatric CD patients.** A1a refer to less than 10 years old, A1b refers to more than 10 years old.

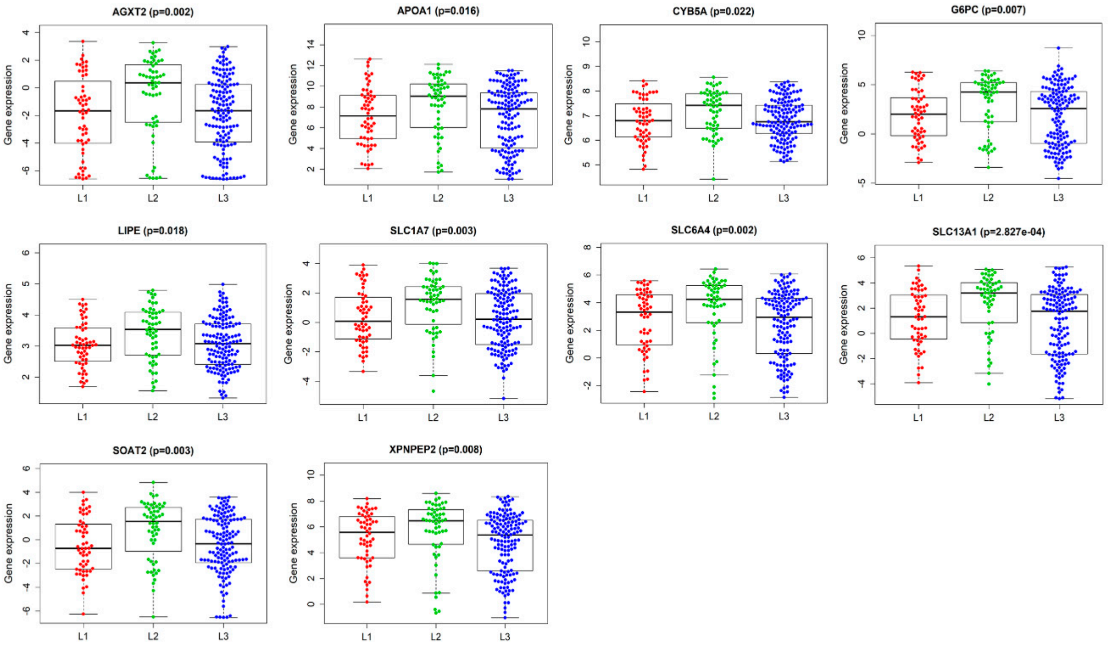


**Figure S2. Expression of 10 hub genes regarding the location-at-diagnosis of pediatric CD**. L1 ileal only, L2 colon only, L3 both ileal and colon.
